# A model of food reward learning with dynamic reward exposure

**DOI:** 10.3389/fncom.2012.00082

**Published:** 2012-10-11

**Authors:** Ross A. Hammond, Joseph T. Ornstein, Lesley K. Fellows, Laurette Dubé, Robert Levitan, Alain Dagher

**Affiliations:** ^1^Center on Social Dynamics and Policy, The Brookings InstitutionWashington, DC, USA; ^2^Montreal Neurological Institute and Hospital, McGill UniversityMontreal, QC, Canada; ^3^Desautels Faculty of Management, McGill UniversityMontreal, QC, Canada; ^4^Department of Psychiatry, University of TorontoToronto, ON, Canada

**Keywords:** reward learning, computational modeling, temporal difference learning, food choice

## Abstract

The process of conditioning via reward learning is highly relevant to the study of food choice and obesity. Learning is itself shaped by environmental exposure, with the potential for such exposures to vary substantially across individuals and across place and time. In this paper, we use computational techniques to extend a well-validated standard model of reward learning, introducing both substantial heterogeneity and dynamic reward exposures. We then apply the extended model to a food choice context. The model produces a variety of individual behaviors and population-level patterns which are not evident from the traditional formulation, but which offer potential insights for understanding food reward learning and obesity. These include a “lock-in” effect, through which early exposure can strongly shape later reward valuation. We discuss potential implications of our results for the study and prevention of obesity, for the reward learning field, and for future experimental and computational work.

## Introduction

Obesity has a complex etiology, with multiple known pathways (Huang and Glass, 2008; Hammond, 2009; Dubé et al., 2010; IOM, 2010, 2012). Considerable evidence suggests the food environment can be an important driver of obesity (Lakdawalla and Philipson, 2009), and that individuals may differ in their propensity to over-consume in response to food cues in the environment (Guerrieri et al., 2008). Some researchers refer to “hedonic hunger”—hunger driven by food cues and the anticipation of food pleasure rather than purely homeostatic caloric needs (Lowe and Butryn, 2007)—underlining the importance of brain reward systems in guiding eating decisions.

We focus on the proposition that preference for high calorie foods, and the inability to resist the appeal of food cues, develops in part through a form of conditioning (Epstein et al., 2007). Conditioning refers to the attribution of incentive properties to previously neutral cues paired with primary rewards, such as food, via *learning* (Frank and Claus, 2006; Samson et al., 2010). Individuals with an enhanced ability to learn from rewards would be more prone to this form of conditioning, and also to the related phenomenon of sensitization, which refers to a progressive increase in the neural and behavioral response to repeated rewards (Robinson and Berridge, 1993). Animal research strongly suggests that inherent differences in the dopamine system promote differential learning about reward-predicting cues, which in turn promotes greater motivation to consume and seek the associated reward in the presence of such cues (Dalley et al., 2005, 2007; Petrovich and Gallagher, 2007; Flagel et al., 2008, 2009; Berridge et al., 2009; Yager and Robinson, 2010; Lovic et al., 2011).

There is considerable evidence that a similar process contributes to human eating behavior and obesity. Obese individuals tend to display a host of personality features and behaviors supportive of a phenotype characterized by increased attraction to high calorie foods, as confirmed by personality questionnaires, laboratory assessments of eating behavior, and functional brain imaging. Some obese individuals: experience greater hedonic responses to sweet or fatty foods (Blundell et al., 2005); have a sensory preference for fat (Mela and Sacchetti, 1991); score higher on questionnaire measures of sensitivity to reward (Davis et al., 2007); work harder in laboratory settings for high calorie snacks (Epstein et al., 2007); demonstrate greater brain activation to food cues as assessed by functional magnetic resonance imaging (Rothemund et al., 2007); are more prone to eating in response to cues and relatively less sensitive to internal homeostatic signals (Herman and Polivy, 2008); exhibit greater activation in the brain areas involved in reward and motivation (Dagher, 2012). Moreover, obese individuals often demonstrate compulsive food seeking behaviors that are reminiscent of drug addiction (Grigson, 2002). The role of food as a reward cue for conditioning, especially via flavor, has also been well studied (Schultz, 1998; Sclafani et al., 2011). In short, there is good evidence that activation of the reward system (e.g., by food cues) is sufficient to drive food consumption beyond homeostatic needs, and thus to promote excess consumption (Petrovich and Gallagher, 2007; Berthoud and Morrison, 2008). Individual differences in the development of the reward system, and the resulting attribution of incentive salience to food, are thus likely to be important drivers of obesity-related behavior.

The model we present in this paper is not intended to be a comprehensive model of eating behavior, but focuses specifically on elucidating the role of reward learning. By excluding other contributing factors such as homeostasis, executive control, and eating norms, we isolate the dynamic effect of reward learning in the context of diverse and changing environmental reward exposure. Our model does not explicitly refer to dopamine, even though its role in learning and sensitization to drugs and foods is not in doubt (Sclafani et al., 2011). Rather we propose that an inherited vulnerability (enhanced reward learning) in conjunction with an environment rich in high calorie foods, can lead to long-lasting neural adaptations that promote over-eating throughout life. We explore the hypothesis that dynamic reward learning can help explain both the importance of early life as a key period in the development of eating behavior and the contradictory evidence surrounding the effect of the food environment on eating behavior and obesity (Morland et al., 2006; Larson et al., 2009; Murakamia et al., 2010).

The learning model used here is a temporal difference learning algorithm (TDL) (Montague et al., 1996; Schultz et al., 1997; Sutton and Barto, 1998). This model is of particular interest as extensive human and animal evidence suggests that TDL signals are carried by dopamine neurons in the brain (Schultz, 1998), and experimental studies have validated this general mathematical model of learning at the individual level in carefully controlled conditions (Montague et al., 1996; Schultz et al., 1997; O'Doherty et al., 2003). In the context of food choice, an individual's environment can strongly shape the consumption choices available, and thus the course of learning. Moreover, the environment to which an individual is exposed may change over time. If TDL is to provide a practical framework for modeling food reward learning, then these considerations must be included. Our primary focus is not to evaluate the effectiveness of the algorithm at achieving appropriate learning in a complex spatial context (as in Tesauro, 1992; Ng et al., 2004; Whiteson et al., 2010), but rather to explore its implications for food choice under heterogeneous dynamic patterns of environmental exposure.

In this paper, we develop an extension of the TDL framework to explicitly model movement across different exposure environments through time. To capture these dynamics and local heterogeneity in environmental exposure, we construct a simulation using agent-based computational modeling (ABM), a framework well-suited to modeling dynamics, learning, and non-random spatial structures (Page, 1999; Axelrod, 2006; Hammond and Axelrod, 2006; Tesfatsion and Judd, 2006). The multi-agent approach also allows for future extensions to the model, such as the incorporation of empirical data on social interactions, food geographies, and additional neurobiological pathways. Reward learning as modeled here can thus be incorporated into a more comprehensive “systems” modeling approach to obesity (Auchincloss and Diez Roux, 2008; Mabry et al., 2008, 2010; Huang et al., 2009; IOM, 2010, 2012; Levy et al., 2011; Hammond and Dube, 2012).

Our results show how differential and dynamic reward exposures can lead to non-trivial differences in the course of learning among individuals. We also demonstrate how early exposure can strongly influence reward learning, and may “lock-in” early experience in a way that shapes later behavior. We begin with the simplest possible model, replicating the expected analytical results from the base TDL formulation, and then sequentially add individual heterogeneity, spatial complexity, and dynamic reward exposures to explore specific hypotheses about the impact of each on reward learning outcomes.

## Materials and methods

### The temporal difference learning framework

In its standard form, the TDL model simulates reward learning via signals of reward-prediction error (which may be signaled in the brain by dopamine). The magnitude of error signaling is represented by the term delta (δ), which is the difference between the actual experienced value of the reward at time *t*, *V*(*t*), and the agent's predicted value of the reward, $\hat{V}(t)$. Predicted value is updated each round according to

(1) $$\hat{V}(t+1)=\hat{V}(t)+\alpha \delta (t),$$

where α is the rate of learning.

In this paper, we adapt this framework to a model of food reward learning. We define a variety of food types, with different reward values associated with consuming them. Each food type *j* has an *intrinsic palatability* (*p*_*j*_). To allow for the possibility of individual heterogeneity in preferences and food reward, our adaptation of the TDL framework permits the “true” *V* associated with each food type to differ between agents. We allow *V* to vary for each agent *i*, based on some multiple of base palatability—beta (β). We refer to β_*ij*_ as agent *i*'s *responsivity* to food *j*. This extension of the standard TDL model is appropriate for modeling situations where reward valuation varies among individuals, as in food choice. Thus:

(2) $$V_{ij}\left(t\right)=\beta_{ij}p_{j}$$

And our modified Equation 1 becomes:

(3) $${\hat{V}}_{ij}\left(t+1\right)={\hat{V}}_{ij}\left(t\right)+\alpha_{i}\left[\beta_{ij}p_{j}-{\hat{V}}_{ij}\left(t\right)\right]$$

We use this formulation of the temporal difference reward learning update rule for the individuals in our stochastic agent-based simulation.

### An initial agent-based TDL model

We begin with a basic framework for agent-based TDL, initially without including any individual heterogeneity or spatial complexity. Our agents are embedded in a food-rich environment, and move about local space consuming food and learning reward values of food types using the TDL rule^1^. Agents eat at a constant rate and homeostatic hunger signals are not modeled, reflecting our focus on food choice based solely on anticipated reward. The “base case” with well-mixed food environment and no individual heterogeneity replicates the expected individual- and population-level learning curves from the standard mathematical formulation of TDL. We then introduce heterogeneity in both individual learning and in local environmental exposure through time, and explore the richer dynamics this generates.

The base model contains two food types: “low” (L) and “high” (H) palatability. Palatability could refer to any feature of a food that causes it to be rewarding, such as energy density. Associated reward values are *p*_*L*_ = 0.6 and *p*_*H*_ = 0.9. We define θ as the ratio of high-to-low palatability (in this base model θ = 1.5). The environment is abstract, and consists of a torus of 100 × 100 cells. Each cell contains two food objects, which are distributed at random—some cells contain two H objects, some contain two L objects, and some contain one of each type. Agents are homogeneous in all key parameters (α,β), and all agents begin with ${\hat{V}}_{ij}(0)=0$. Each period of the simulation has three steps:

1. All agents move (in random order) to a randomly chosen available cell adjacent to their current location in the abstract food environment.
2. All agents (in random order) consume a single food item from the two available options in the cell they currently occupy. If the cell contains HH or LL, no decision is required—agents simply consume one object of the only available food type. If the cell contains HL, agents use current internal expected valuations ${\hat{V}}_{ij}(t)$ and choose the food type with the higher value. We introduce a small amount of noise, in the form of a probability ε (0.05 unless otherwise noted) that the agent picks the lower-valued food type (and picks the higher-valued food type with probability 1−ε).
3. All agents update ${\hat{V}}_{ij}(t)$ using the individual TDL rule identified in Equation 3 above.

**Figure 1:**
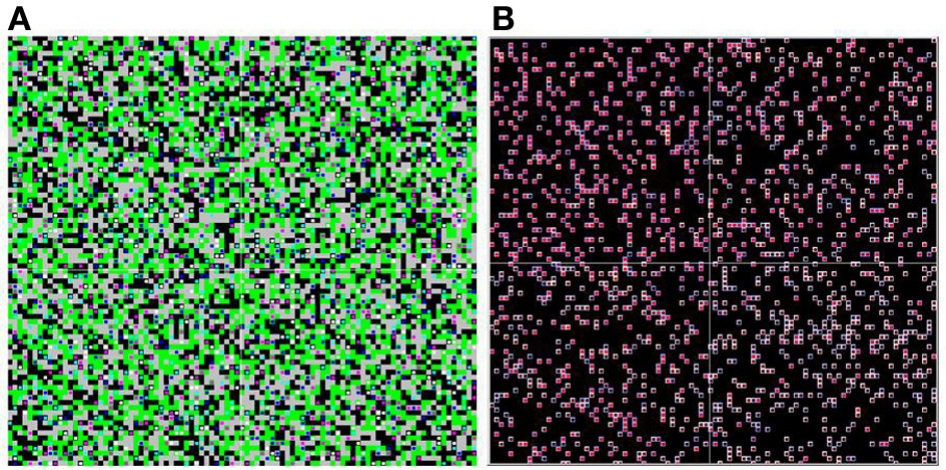
**Food environment and heat map.** The spatial environment is displayed as if viewed “from overhead.” Two versions are shown; the panel on the left shows the food environment and distribution of agents, while the panel on the right shows the internal (reward learning) states of the agents. In the left panel view (**A**), the color of each square in the environment represents the mixture of food objects at that location (black = HH, green = LL, gray = HL). The colored dots represent individual agents, showing their location. In the right panel view (**B**), the environmental information is suppressed, and the dot colors represent the internal reward valuations for each individual agent. The inside color represents ${\hat{V}}_{H}$, and goes from black (H not learned) to bright red (H fully learned). The outside color represents ${\hat{V}}_{L}$, and goes from white (L not learned) to dark blue (L fully learned).

This process is repeated until the simulation reaches an equilibrium (no agent is still changing its reward valuations). In the base case, this generally occurs within a few hundred iterations. The simulation records the process of learning through time for all individuals [e.g., ${\hat{V}}_{ij}(t)$ for all *i*,*j*,*t*], and these are displayed on an animated spatial map (Figure 1) and analyzed statistically to produce population learning curves.

**Figure 2:**
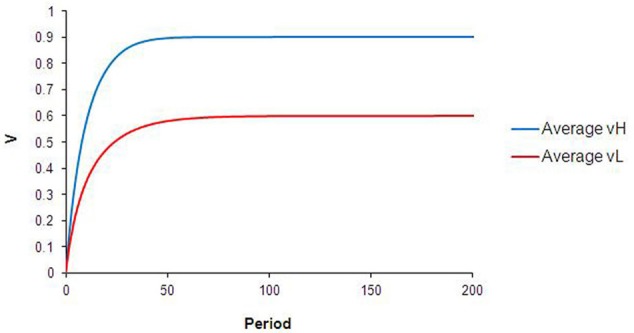
**Results from standard case.** The curves in this chart describe the average ${\hat{V}}_{H}$ and ${\hat{V}}_{L}$ values for the agent population during all runs of the base case simulation.

With no heterogeneity in either individual learning parameter (α_*i*_ = 0.4, β_*ij*_ = 1 for all *i*) and with a well-mixed spatial distribution of food types, the internal reward valuations for all agents converge rapidly to “intrinsic” (*p*) values for each type of food. Figure 2 shows the population-level average learning curves for both H and L food that result from the standard case simulation. These correspond to the learning curves expected from the standard TDL equations.

## Results

### Individual heterogeneity in learning

We now relax the assumption of individual homogeneity, allowing key learning parameters to vary. This allows us to explore how individual heterogeneity affects learning through time (at both individual and population levels). Agents now vary in both learning rate (α_*i*_) and food-type responsivity (β_*ij*_) for the H-food type (we do not model L-type responsivity). Each parameter is given a low variant and high variant to allow simple comparison. This results in four agent “types”: (1) fast learners highly responsive to H-food (α_*i*_ = 0.4, β_*iH*_ = 2.0); (2) fast learners with low responsivity to H-food (α_*i*_ = 0.4, β_*iH*_ = 0.5); (3) slow learners highly responsive to H-food (α_*i*_ = 0.1, β_*iH*_ = 2.0); (4) slow learners with low responsivity to H-food (α_*i*_ = 0.1, β_*iH*_ = 0.5).

The agent population is evenly divided between these four types, and agents are distributed in random initial locations in space (as shown in Figure 1A). The mixture of food objects in each cell is also distributed at random as before, and the simulation proceeds with the same three steps per round.

**Figure 3:**
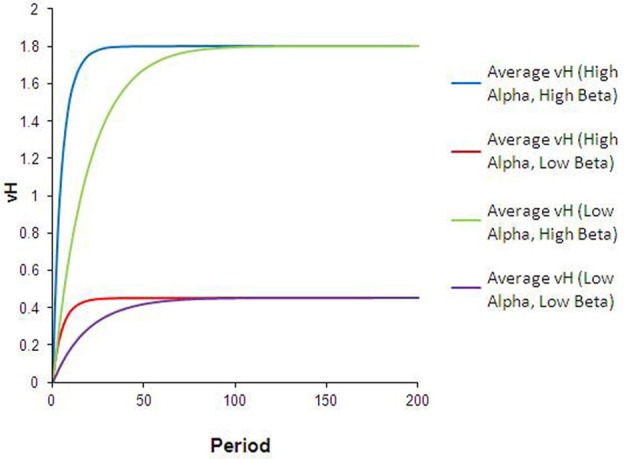
**Results from heterogeneous learning parameters.** The curves in this chart describe the average ${\hat{V}}_{H}$ and ${\hat{V}}_{L}$ for each subpopulation during every run of the simulation in this configuration. Differences in alpha lead to convergence at *different rates* to the same ${\hat{V}}_{H}$ (blue vs. green lines), while differences in beta result in convergence to *different levels* of ${\hat{V}}_{H}$ (green vs. purple lines).

The dynamics that result from this type of heterogeneity are intuitive. As illustrated in Figure 3, the reward valuation for all agents converges to β_*ij*_*p*_*j*_, and at a faster rate for agents with high-α than for agents with low-α. Differences in α (between types 1 and 3, or 2 and 4) lead to convergence to the same final *V*_*H*_ but at different rates. Differences in β (between types 1 and 2, or 3 and 4) lead to convergence to different ending *V*_*H*_. The qualitative comparisons are robust to variation in the specific values of α and β used. Although not unexpected, these results are significant. Interpreted in the context of food choice, differences in learning rates (or perceived reward valuation) could translate into non-trivial calorie surpluses for high-α or high-β agents.

### Heterogeneity in spatial exposure

**Figure 4:**
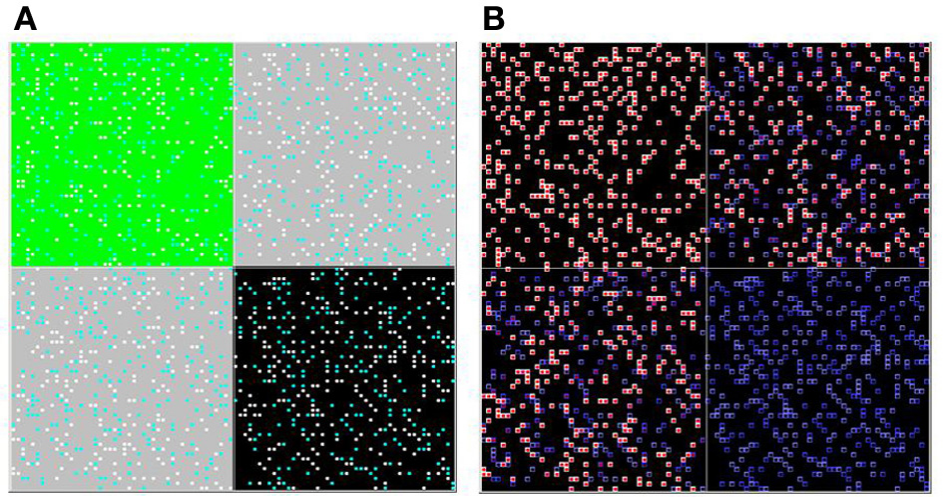
**Spatial heterogeneity.** Agents are randomly seeded into one of four quadrants, and cannot move between them. The upper-left quadrant is entirely high-palatable, the lower-right is entirely low-palatable, and the remaining two quadrants are mixed. The simulation is shown here mid-way through a run. Two versions of the overhead view (of all four quadrants) are shown **(A** and **B)**, with coloring as described in Figure 1.

Next, we incorporate *spatial heterogeneity* into the model (initially in a very restricted sense, so that we can conduct rigorous tests of the model's assumptions). The space is divided into four distinct regions; agents can move freely within their own region but cannot cross into adjacent regions. The first region contains only high-palatable food items (upper-left in Figure 4A), the second contains only low-palatable foods (lower-right in Figure 4A), and the final two regions contain mixed high- and low-palatable foods.

**Figure 5:**
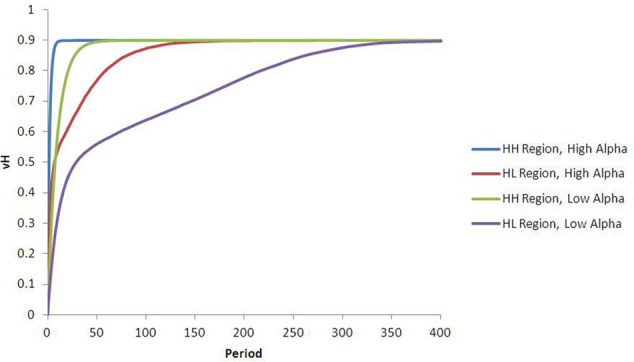
**Fifteen run average results: heterogeneous agents and distinct regions.** Agents in different regions learn the value of food H at different rates. In this case, agents in Region LL never learn the value of food H, agents in Region HH learn it quickly (it is the only food available), and agents in Region HL learn slower on average, due to lock-in. 15-run standard errors never exceed 0.01, and are not shown.

Agents in the high-palatable region learn the value of H as before, and agents in the low-palatable region do not learn the value of H at all. But agents in the mixed region, even though they have the same learning parameters as those in the first region, learn at a slower rate. This results from “lock-in” of early choices; half of the subjects choose the low-palatable food initially, and then will only attempt to learn the value of H with probability ε. The effect is clearly evident even mid-way through a simulation run, as illustrated in the upper-right and lower-left quadrants of the heat map in Figure 4B. The result is a lower effective learning rate (see Figure 5).

### Reward learning with movement and spatial dynamics

Our core motivation in extending and applying the TDL framework is to explore the implications of reward learning dynamics with changing environmental exposures. To gain further insight into these dynamics, we now add to our model the potential for individual movement *across* environmental contexts over time. Rather than have agents learn in a static environment, we introduce transitions across regions during the learning process. This allows us to determine whether the “lock-in” of initial exposure demonstrated above (Figure 5) persists when agents are not restricted to a single uniform environment.

**Figure 6:**
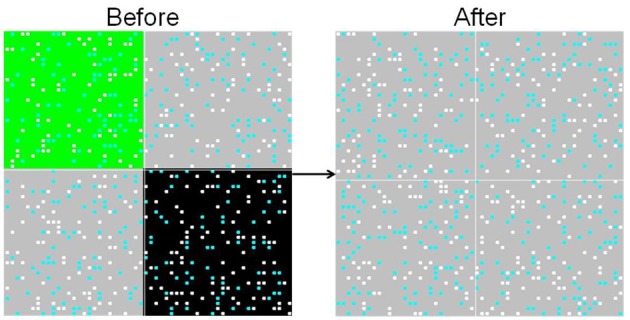
**Movement from pure initial exposure conditions.** To simulate movement, the food environments in the HH and LL quadrants are switched to HL during the course of the run.

As illustrated in Figure 6, the movement experiment has two phases: (1) All agents are initialized in one of three regions (HH, LL, or HL). Initial learning occurs in this environment as before. (See *Before* in Figure 6). We continue this phase until agents in the HH region have completed learning. (2) Every agent is moved to a mixed (HL) environment. (See *After* in Figure 6). Of particular interest is the time taken by agents initially exposed to the low-palatable environment to learn the value of the high-palatable food following the move.

**Figure 7:**
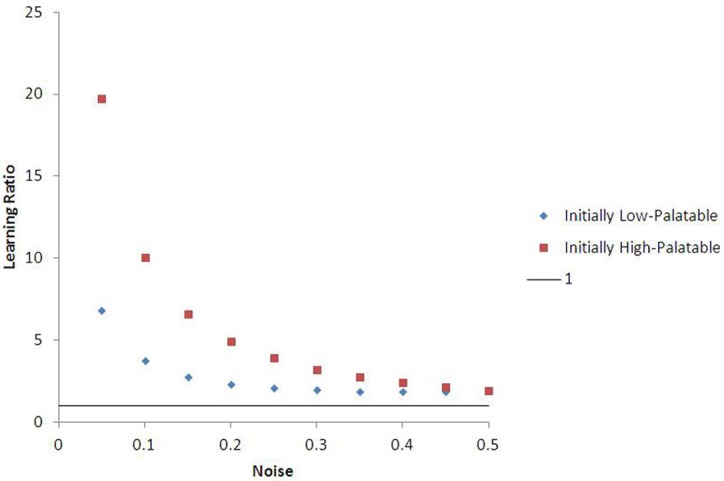
**Learning ratio as a function of noise, with 15-run standard error bars.** The chart describes the ratio between the learning rate for an agent's second food vs. the same agent's first food (θ = 1.5, alpha = 0.4). The data suggest that lock-in is quite robust to changes in noise, as the learning ratio is greater than one for all values of epsilon. Agents who begin learning in the initially high-palatable region experience greater lock-in magnitude than those in the initially low-palatable region, though this effect diminishes as noise increases.

Outcomes in these experiments can be affected by the amount of “noise” in food choice (default ε = 0.05) and the palatability ratio (θ) between low and high value food (default θ = 1.5). Nonetheless, for a broad range of parameterizations, there are substantial differences in “effective” learning rate (and choice behavior over long periods of time) conditioned by early exposure environment (see Figure 7). For instance, agents learning the value of high-palatability food take on average 6.8 times longer to do so if they were originally conditioned in a low-palatability environment than those originally seeded in the high-palatability environment (at ε = 0.05, θ = 1.5). This ratio is 19.7 for agents learning the value of low-palatability food. More generally, agents on average learn their initial food value at a faster rate than the second food they are exposed to (after moving), regardless of which is low- or high-palatable. This consistent lock-in effect is quite robust to noise (the learning ratio remains above 1 even at ε = 0.50, as Figure 7 shows). Agents that initiate learning in the high-palatability region exhibit greater levels of lock-in than those that initiate learning in the low-palatability region. This difference is magnified by higher values of θ, and diminishes as noise increases.

### Robustness to mixed initial exposure conditions

The “lock-in” effects described above were based on movement from a homogeneous environment to a mixed environment. Here we explore robustness of the result to initial agent environments that are more heterogeneous. Does lock-in require a heavily-skewed initial environment? This question is of high interest for the study of food reward, because there is substantial heterogeneity in the early exposure environments faced by human learners.

**Figure 8:**
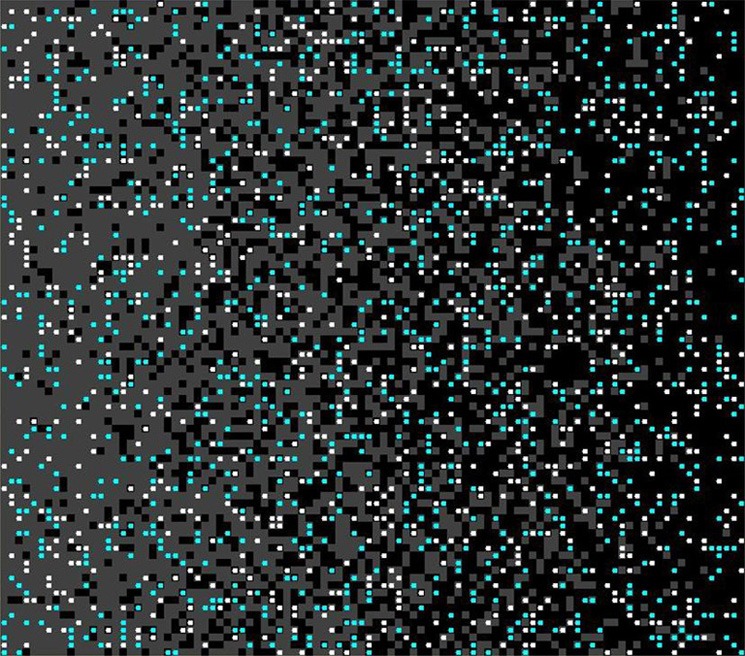
**Gradient space.** Gray is low-palatable (*p* = 0.6), and black is high-palatable (*p* = 0.9).

To test this possibility, we embed the agents in a *gradient* space, with 100% high-palatable food on one edge of the lattice, 100% low-palatable food on the other edge, and a smooth gradation of levels in between. (This map has the useful property that an agent on *x*-position *x*_*i*_ has a *x*_*i*_% probability that its host cell contains high-palatable food; See Figure 8). In this formulation, agents choose from the set of food on their cell or the surrounding 8 neighbors (choosing the food with highest reward valuation with probability 1−ε, and a random food in their neighborhood with probability ε).

**Figure 9:**
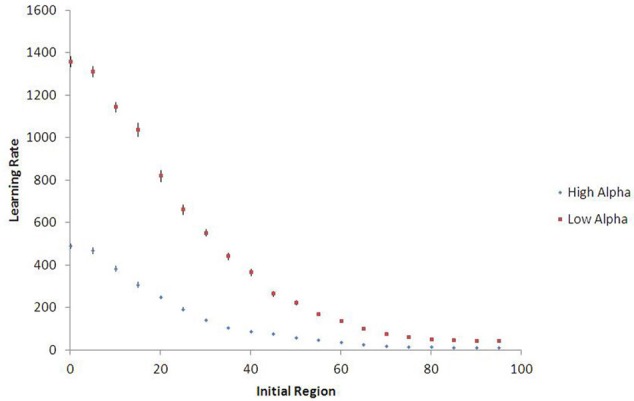
**Learning rates in continuous space, with 15-run standard error bars.** Agents who begin learning in regions with higher proportions of high-palatable food experience less lock-in and learn its value more quickly (θ = 1.5). Results shown are population averages across 15 runs of the stochastic simulation.

**Figure 10:**
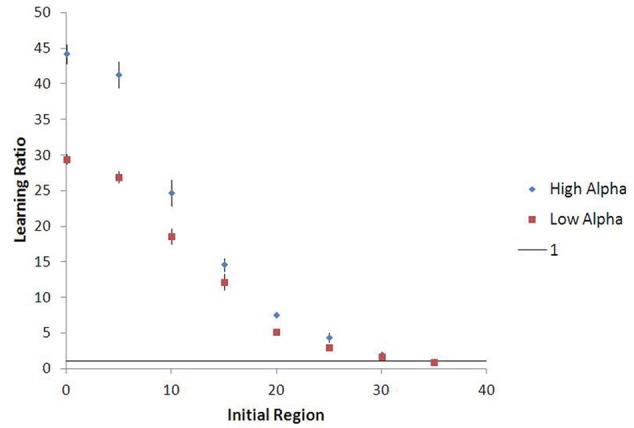
**Lock-in persistence in continuous space, with 15-run standard error bars.** The model yields learning ratios greater than one even for agents seeded in initial food environments with substantial mixtures of both food types (θ = 1.5). Results shown are averages across 15 runs of the stochastic simulation.

Figure 9 shows that agents who begin in a more low-palatable region take longer on average to learn the value of the high-palatable food, as expected. Because this new variant also provides a continuous-space analog to the movement experiments, we are able to demonstrate persistent lock-in (represented by ratio > 1 of time to learn *V*_*H*_ vs. *V*_*L*_) even when the initial food environment contains a substantial proportion of both food types (see Figure 10).

## Discussion

The model we present here introduces population spatial dynamics and substantial individual and spatial heterogeneity into a well-supported model of food reward learning. We used the computational technique of agent-based modeling to first replicate findings from the standard TDL formulation, and then apply it to a stylized spatial context of changing environmental exposures through time. This extended model uncovers a variety of agent behaviors which are not evident from the traditional formulation, but which offer insights of potentially high relevance to food reward learning and obesity. These include a “lock-in” effect, through which early exposure can strongly shape later reward valuation.

Our results offer new insights into two important areas of the existing obesity literature—the role of the food environment and the early childhood development of eating behavior. Empirical studies of the relationship between objective measures of the food environment and eating behavior, overweight, and obesity have found very mixed results. For example, two recent systematic review papers (Casey et al., 2011; Caspi et al., 2012) identified one set of studies showing strong and statistically significant relationships, other studies showing no relationships, and still others showing weak or mixed correlations. Both review papers call for further research to explain the conflicting evidence. Our central result (the “lock-in” effect) provides one potential explanation by demonstrating that *early* exposures may shape reward learning and food preferences more strongly than *current* exposures. All 63 empirical studies covered in the review papers examine correlations between *current* food environment and *current* eating or weight; our model suggests the field may need to take into account changing environmental exposures through time and across lifecourse development instead^2^.

Our results also help explain the importance of early childhood in the development of obesity. A large body of experimental and theoretical work illustrates how adult health behavior can trace its roots to neurological and physiological development during childhood (Champagne and Meaney, 2001; Gillman, 2005), and early childhood may be an especially important developmental window for obesity (McMillan and Robinson, 2005; Nader et al., 2012). The “lock-in” effect described in this paper represents one candidate mechanism through which this process may occur. Pending empirical testing of our model in future work, studying food reward learning and choice in this way has the potential to inform novel prevention and treatment approaches for the ongoing obesity epidemic. Identification of key developmental windows during which exposure to healthy (or unhealthy) food has the strongest effect on long-term appetitive behavior would provide an important focus for prevention efforts. Consideration of heterogeneity and dynamic patterns in reward exposure may also allow opportunities for more focused prevention.

Beyond the immediate implications of our results for the study of food choice and obesity, the model of reward learning we have presented here can serve as a foundation for future work extending the computational approach to other neurobiological determinants of eating behavior, and for experimental work aimed at deepening our understanding of food reward learning. Capturing the complexity of eating behavior and obesity is likely to require models that include multiple pathways and mechanisms. We believe that reward learning under dynamic environmental exposure, as modeled here, will be an important component of this type of comprehensive modeling approach.

### Conflict of interest statement

The authors declare that the research was conducted in the absence of any commercial or financial relationships that could be construed as a potential conflict of interest.

## Footnotes

^1^The food environment in the model is abstract, representing exposure to food choices whether in the physical environment, the home environment, or elsewhere. Similarly, movement of the individuals is stylized, following a standard convention in multi-agent simulation for modeling heterogeneous and changing environmental exposures.

^2^This hypothesis should be directly testable with the collection of additional evidence. It is consistent with some recent evidence suggesting stronger correlations between children's environment and eating/weight than between parent's environment and eating/weight (Saelens et al., 2012).
